# 4′,4′,6′,6′-Tetra­chloro-2-(6-methyl­pyridin-2-yl)-1*H*,2*H*-spiro­[naphtho­[1,2-*e*][1,3,2]oxaza­phosphinine-3,2′-[1,3,5,2,4,6]tri­aza­triphosphinine]

**DOI:** 10.1107/S1600536813012348

**Published:** 2013-05-11

**Authors:** Muhammet Işıklan, Ömer Sonkaya, Tuncer Hökelek

**Affiliations:** a Department of Chemistry, Kırıkkale University, 71450, Kırıkkale, Turkey; b Department of Physics, Hacettepe University, 06800 Beytepe, Ankara, Turkey

## Abstract

The title compound, C_17_H_14_Cl_4_N_5_OP_3_, is a spiro-phosphazene derivative with bulky naphthalene and pyridine rings. The phosphazene and the six-membered N/O rings are in flattened-boat and twisted-boat conformations, respectively. The naphthalene ring system and the pyridine ring are oriented at a dihedral angle of 18.06 (8)°. In the crystal, weak π–π stacking between the pyridine rings and between the pyridine rings and the naphthalene ring system [centroid–centroid distances = 3.594 (2) and 3.961 (2) Å, respectively] occur. Weak C—H⋯π inter­actions are also observed. These interactions link the molecules into a three-dimensional supramolecular network.

## Related literature

For products from the reaction of N_3_P_3_Cl_6_ with bifunctional reagents, see: Beşli *et al.* (2006 ▶). For N/O donor type bifunctional reagents used for the reaction of hexa­chloro­cyclo­triphosphazene giving *spiro* derivatives, see: Beşli *et al.* (2007 ▶); Sournies *et al.* (1997 ▶); Deutsch & Shaw (1990 ▶); Işıklan *et al.* (2010 ▶); İlter *et al.* (2004 ▶, 2007 ▶); Tercan *et al.* (2004 ▶). For industrial applications of cyclo­phosphazenes, see: Omotowa *et al.* (2004 ▶); Barbera *et al.* (2005 ▶); Schneider *et al.* (1999 ▶); Ding *et al.* (2005 ▶); Allcock (2006 ▶); Xu *et al.* (2006 ▶); Allcock & Wood (2006 ▶); Li *et al.* (2004 ▶); Singh *et al.* (2006 ▶); Greish *et al.* (2005 ▶). For bond-length data, see: Allen *et al.* (1987 ▶). For the standard compound, N_3_P_3_Cl_6_, see: Bullen (1971 ▶). For ring-puckering parameters, see: Cremer & Pople (1975 ▶).
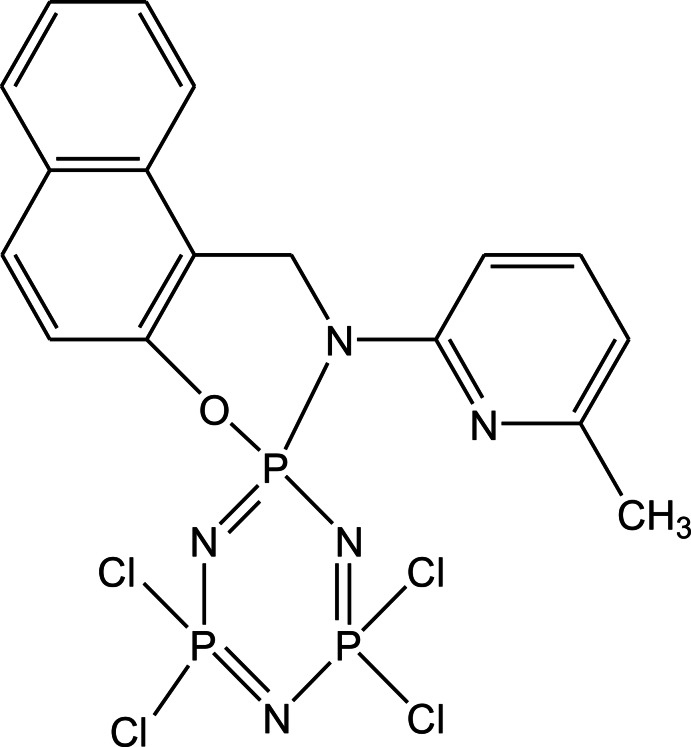


## Experimental

### 

#### Crystal data

C_17_H_14_Cl_4_N_5_OP_3_
*M* *_r_* = 539.04
Orthorhombic, 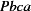

*a* = 21.7784 (5) Å
*b* = 7.8573 (3) Å
*c* = 25.1034 (5) Å
*V* = 4295.7 (2) Å^3^
*Z* = 8
Mo *K*α radiation
μ = 0.80 mm^−1^
*T* = 100 K
0.38 × 0.28 × 0.08 mm

#### Data collection

Bruker Kappa APEXII CCD area-detector diffractometer
Absorption correction: multi-scan (*SADABS*; Bruker, 2005 ▶) *T*_min_ = 0.752, *T*_max_ = 0.939
22083 measured reflections
5297 independent reflections
3446 reflections with *I* > 2σ(*I*)
*R*_int_ = 0.070

#### Refinement

*R*[*F* ^2^ > 2σ(*F* ^2^)] = 0.048
*wR*(*F* ^2^) = 0.129
*S* = 1.01
5297 reflections
272 parameters
H-atom parameters constrained
Δρ_max_ = 0.73 e Å^−3^
Δρ_min_ = −0.77 e Å^−3^

### 

Data collection: *APEX2* (Bruker, 2007 ▶); cell refinement: *SAINT* (Bruker, 2007 ▶); data reduction: *SAINT*; program(s) used to solve structure: *SHELXS97* (Sheldrick, 2008 ▶); program(s) used to refine structure: *SHELXL97* (Sheldrick, 2008 ▶); molecular graphics: *ORTEP-3 for Windows* (Farrugia, 2012 ▶); software used to prepare material for publication: *WinGX* (Farrugia, 2012 ▶) and *PLATON* (Spek, 2009 ▶).

## Supplementary Material

Crystal structure: contains datablock(s) I, global. DOI: 10.1107/S1600536813012348/xu5701sup1.cif

Structure factors: contains datablock(s) I. DOI: 10.1107/S1600536813012348/xu5701Isup2.hkl

Supplementary material file. DOI: 10.1107/S1600536813012348/xu5701Isup3.cml

Additional supplementary materials:  crystallographic information; 3D view; checkCIF report

## Figures and Tables

**Table 1:** Selected bond lengths (Å)

P1—O1	1.580 (2)
P1—N1	1.596 (3)
P1—N3	1.598 (3)
P1—N4	1.665 (3)
P2—N1	1.571 (3)
P2—N2	1.580 (3)
P3—N2	1.581 (3)
P3—N3	1.571 (3)

**Table 2:** Hydrogen-bond geometry (Å, °) *Cg*3 is the centroid of the C8-benzene ring.

*D*—H⋯*A*	*D*—H	H⋯*A*	*D*⋯*A*	*D*—H⋯*A*
C7—H7⋯*Cg*3^i^	0.95	2.82	3.507 (3)	130

Symmetry code: (i) 
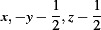
.

**Fig. 1.**
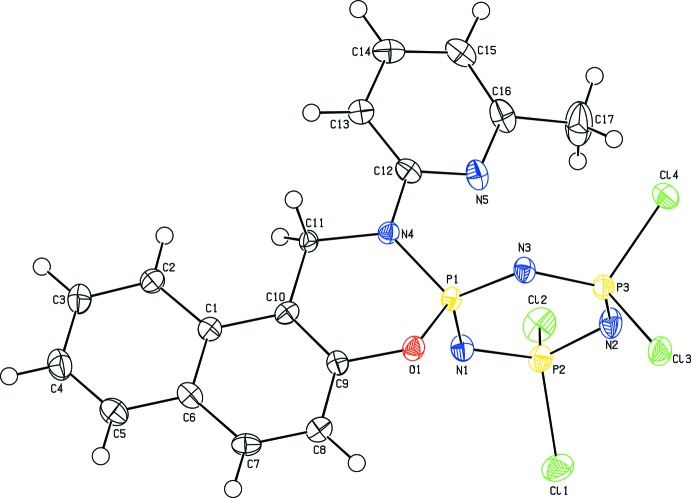
The molecular structure of the title molecule, with the atom-numbering scheme. Displacement ellipsoids are drawn at the 50% probability level.

**Fig. 2.**
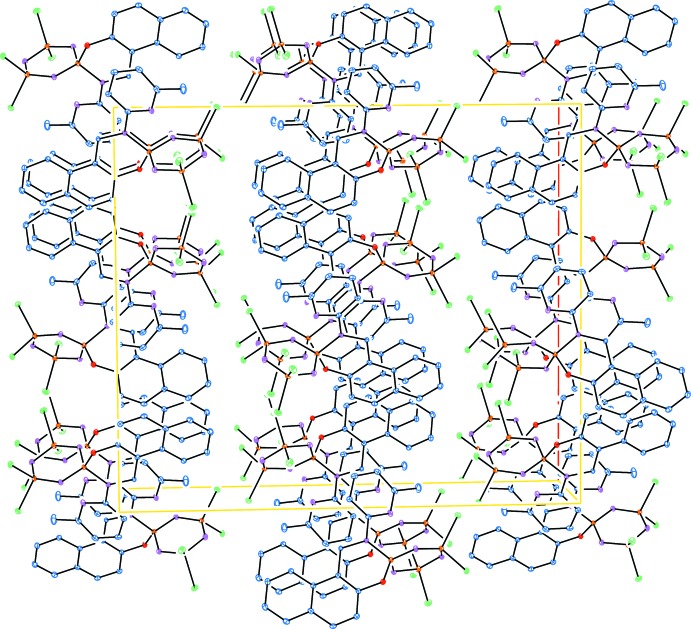
A view along the b axis of the crystal packing of the title compound (a axis vertical; c axis horizontal). Hydrogen atoms have been omitted for clarity.

## supplementary crystallographic information

### Comment

Cyclophosphazenes are known as inorganic heterocyclic rings containing an (N=P*X*_2_)_n_ repeating unit. Hexachlorocyclotriphosphazene, N_3_P_3_Cl_6_, can also be considered as a trimer, which can be obtained by the reaction of phosphorus pentachloride and ammonium chloride. It has been observed that reactions of N_3_P_3_Cl_6_ with bifunctional reagents can afford *spiro*-, *ansa*-, *bino*- and open chain (dangling) products (Beşli *et al.*, 2006). Reaction of hexachlorocyclotriphosphazene with N/O donor type bifunctional reagents such as aminoalcohols (Beşli *et al.*, 2007; Sournies *et al.*, 1997; Deutsch & Shaw, 1990), aminophenols (Işıklan *et al.*, 2010; İlter *et al.*, 2007) and aminonaphthols (İlter *et al.*, 2004; Tercan *et al.*, 2004) predominantly gave *spiro* derivatives. Cyclophosphazenes have found industrial applications such as in the production of ionic liquids (Omotowa *et al.*, 2004), liquid crystals (Barbera *et al.*, 2005), dendrimers having chiral ligands for asymmetric catalysis (Schneider *et al.*, 1999), flame retardants (Ding *et al.*, 2005), advanced elastomers (Allcock, 2006), rechargeable lithium batteries and polymer electrolytes (Xu *et al.*, 2006; Allcock & Wood, 2006), non-linear optics (Li *et al.*, 2004), biomedical mambranes (Singh *et al.*, 2006), and biomedical materials including synthetic bones (Greish *et al.*, 2005). The present study was undertaken to ascertain the crystal structure of the title compound.

In the title compound, (Fig. 1), the phosphazene ring (*A*) is in flattened-boat conformation [φ = 31.7 (9)° and θ = 101.8 (8)°] having total puckering amplitude Q_T_ of 0.137 (2) Å (Cremer & Pople, 1975). Atoms N1, N2 and N3 are displaced from the plane through the P atoms by 0.112 (3), -0.154 (3) and -0.029 (3) Å, respectively. As expected, the naphthalene and pyridine rings are planar, and they are oriented at a dihedral angle of 18.06 (8)°. Ring *B* (P1/O1/C9—C11/N4) is in twisted-boat conformation [φ = 147.1 (7)° and θ = 96.9 (8)°] having total puckering amplitude Q_T_ of 0.482 (7) Å.

In the phosphazene ring, the P—N bond lengths are in the range of 1.571 (3)–1.598 (3) Å [average value is 1.583 (3) Å] (Table 1), exhibiting a regular variation with distances from P1: P1—N1 ≈ P1—N3 〉 P2—N2 ≈ P3—N2 〉 P2—N1 ≈ P3—N3, and showing double-bond character. However, the exocyclic P1—N4 bond [1.665 (3) Å] is at the lower limit of the single bond length. In the phosphazene compounds, the P—N and P═N bonds are generally in the ranges of 1.628–1.691 and 1.571–1.604 Å, respectively (Allen *et al.*, 1987). The shortening in the P1—N4 bond is probably due to electron transfer from N4 to the phosphazene ring.

In the phosphazene ring, the endocyclic N1—P1—N3 angle [117.31 (14)°] is decreased and the exocyclic O1—P1—N4 angle [102.37 (12)°] is increased, with electron donation and withdrawal by the substituents, relative to the 'standard compound' N_3_P_3_Cl_6_ (Bullen, 1971). In the latter compound, the corresponding angles are 118.3, 118.5, 101.2 and 101.6 °, respectively.

The P1—N1—P1, P2—N2—P3 and P1—N3—P3 angles are 121.72 (17), 119.79 (17) and 120.78 (17) °, respectively; P2—N2—P3 and P1—N3—P3 are decreased, with electron donation and withdrawal by the N_3_P_3_ ring. They can be compared with the average value reported for N_3_P_3_Cl_6_, *viz*. 121.4 (3)°.

In the crystal, molecules are stacked nearly parallel to (101) [Fig. 2]. Two weak C—H···π interactions (Table 2) and two weak π–π contacts between the pyridine rings and between the pyridine and naphthalene rings, *Cg*5—*Cg*5^i^ and *Cg*5—*Cg*3^ii^ [symmetry codes: (i) - *x*, - *y*, 1 - *z*, (ii) - *x*, 1 - *y*, 1 - *z*, where *Cg*3 and *Cg*5 are the centroids of the rings (C1/C6—C10) and (N5/C12—C16), respectively] may stabilize the structure, with centroid-centroid distances of 3.594 (2) and 3.961 (2) Å, respectively.

### Experimental

Hexachlorocyclotriphosphazene (1.00 g, 2.88 mmol) in dry THF (100 ml) was introduced into a 250 ml three-necked round-bottomed flask. One equivalent of 1-[(6-methylpyridin-2-ylamino)methyl]naphthalene-2-ol (0.76 g, 2.88 mmol) and triethylamine (5 ml, 36.00 mmol) were dissolved in dry THF (50 ml) and placed in an addition funnel. The 1-[(6-methylpyridin-2-ylamino)methyl]- naphthalene-2-ol and triethylamine solution was added dropwise to the hexachlorocyclotriphosphazene solution and allowed to react for 24 h. The progress of the reaction was monitored by TLC. The precipitated triethylamine hydrochloride was filtered off. The solvent was evaporated and the product was purified through a silica gel column with a mobile phase of CHCl_3_. The oily product was recrystallized in n-hexane [m.p. 495.5 K, yield: 0.70 g, 45%].

### Refinement

H atoms were positioned geometrically with C—H = 0.95, 0.99 and 0.98 Å for aromatic, methylene and methyl H atoms, and constrained to ride on their parent atoms, with *U*_iso_(H) = k × *U*_eq_(C), where k = 1.5 for methyl H-atoms and k = 1.2 for all other H-atoms.

### Crystal data

**Table:**

C_17_H_14_Cl_4_N_5_OP_3_	*F*(000) = 2176
*M**_r_* = 539.04	*D*_x_ = 1.667 Mg m^−^^3^
Orthorhombic, *P**b**c**a*	Mo *K*α radiation, λ = 0.71073 Å
Hall symbol: -P 2ac 2ab	Cell parameters from 2741 reflections
*a* = 21.7784 (5) Å	θ = 2.5–26.6°
*b* = 7.8573 (3) Å	µ = 0.80 mm^−^^1^
*c* = 25.1034 (5) Å	*T* = 100 K
*V* = 4295.7 (2) Å^3^	Plate, colorless
*Z* = 8	0.38 × 0.28 × 0.08 mm

### Data collection

**Table:**

Bruker Kappa APEXII CCD area-detector diffractometer	5297 independent reflections
Radiation source: fine-focus sealed tube	3446 reflections with *I* > 2σ(*I*)
Graphite monochromator	*R*_int_ = 0.070
φ and ω scans	θ_max_ = 28.3°, θ_min_ = 2.5°
Absorption correction: multi-scan (*SADABS*; Bruker, 2005)	*h* = −25→29
*T*_min_ = 0.752, *T*_max_ = 0.939	*k* = −10→6
22083 measured reflections	*l* = −31→33

### Refinement

**Table:**

Refinement on *F*^2^	Primary atom site location: structure-invariant direct methods
Least-squares matrix: full	Secondary atom site location: difference Fourier map
*R*[*F*^2^ > 2σ(*F*^2^)] = 0.048	Hydrogen site location: inferred from neighbouring sites
*wR*(*F*^2^) = 0.129	H-atom parameters constrained
*S* = 1.01	*w* = 1/[σ^2^(*F*_o_^2^) + (0.0611*P*)^2^ + 1.3984*P*] where *P* = (*F*_o_^2^ + 2*F*_c_^2^)/3
5297 reflections	(Δ/σ)_max_ < 0.001
272 parameters	Δρ_max_ = 0.73 e Å^−^^3^
0 restraints	Δρ_min_ = −0.77 e Å^−^^3^

### Special details

**Table:**

Geometry. All e.s.d.'s (except the e.s.d. in the dihedral angle between two l.s. planes) are estimated using the full covariance matrix. The cell e.s.d.'s are taken into account individually in the estimation of e.s.d.'s in distances, angles and torsion angles; correlations between e.s.d.'s in cell parameters are only used when they are defined by crystal symmetry. An approximate (isotropic) treatment of cell e.s.d.'s is used for estimating e.s.d.'s involving l.s. planes.
Refinement. Refinement of *F*^2^ against ALL reflections. The weighted *R*-factor *wR* and goodness of fit *S* are based on *F*^2^, conventional *R*-factors *R* are based on *F*, with *F* set to zero for negative *F*^2^. The threshold expression of *F*^2^ > σ(*F*^2^) is used only for calculating *R*-factors(gt) *etc*. and is not relevant to the choice of reflections for refinement. *R*-factors based on *F*^2^ are statistically about twice as large as those based on *F*, and *R*- factors based on ALL data will be even larger.

### Fractional atomic coordinates and isotropic or equivalent isotropic displacement parameters (Å^2^)

**Table:**

	*x*	*y*	*z*	*U*_iso_*/*U*_eq_	
Cl1	0.23736 (4)	0.09286 (12)	0.33683 (4)	0.0310 (2)	
Cl2	0.12400 (4)	−0.12370 (11)	0.36507 (4)	0.0315 (2)	
Cl3	0.12654 (4)	0.54678 (12)	0.27056 (3)	0.0268 (2)	
Cl4	0.00013 (4)	0.37802 (12)	0.28234 (3)	0.0277 (2)	
P1	0.10765 (4)	0.37524 (10)	0.42559 (3)	0.01570 (18)	
P2	0.14946 (4)	0.11972 (11)	0.35899 (3)	0.0203 (2)	
P3	0.08161 (4)	0.38196 (11)	0.31822 (3)	0.01836 (19)	
O1	0.15089 (10)	0.5220 (3)	0.44668 (8)	0.0188 (5)	
N1	0.14830 (12)	0.2097 (3)	0.41488 (10)	0.0195 (6)	
N2	0.11206 (13)	0.2007 (4)	0.31138 (11)	0.0243 (7)	
N3	0.07526 (12)	0.4618 (3)	0.37531 (10)	0.0183 (6)	
N4	0.06038 (12)	0.3449 (3)	0.47683 (10)	0.0161 (6)	
N5	0.00488 (13)	0.1705 (3)	0.42067 (11)	0.0220 (6)	
C1	0.15699 (15)	0.5167 (4)	0.59375 (12)	0.0170 (7)	
C2	0.12413 (15)	0.4649 (4)	0.63968 (12)	0.0197 (7)	
H2	0.0861	0.4068	0.6357	0.024*	
C3	0.14637 (16)	0.4972 (4)	0.68968 (13)	0.0241 (8)	
H3	0.1242	0.4585	0.7199	0.029*	
C4	0.20163 (17)	0.5872 (5)	0.69686 (14)	0.0279 (8)	
H4	0.2163	0.6104	0.7317	0.033*	
C5	0.23409 (16)	0.6410 (4)	0.65326 (14)	0.0248 (8)	
H5	0.2713	0.7021	0.6581	0.030*	
C6	0.21268 (15)	0.6066 (4)	0.60098 (13)	0.0202 (7)	
C7	0.24658 (15)	0.6603 (4)	0.55567 (13)	0.0213 (7)	
H7	0.2839	0.7211	0.5606	0.026*	
C8	0.22672 (15)	0.6265 (4)	0.50547 (13)	0.0203 (7)	
H8	0.2499	0.6612	0.4753	0.024*	
C9	0.17088 (14)	0.5387 (4)	0.49938 (12)	0.0167 (6)	
C10	0.13573 (14)	0.4829 (4)	0.54063 (12)	0.0164 (6)	
C11	0.07536 (14)	0.3917 (4)	0.53259 (11)	0.0147 (6)	
H11A	0.0758	0.2865	0.5543	0.018*	
H11B	0.0420	0.4651	0.5464	0.018*	
C12	0.00836 (14)	0.2393 (4)	0.46936 (13)	0.0184 (7)	
C13	−0.03482 (15)	0.2132 (4)	0.50891 (13)	0.0182 (7)	
H13	−0.0292	0.2574	0.5438	0.022*	
C14	−0.08672 (15)	0.1197 (4)	0.49558 (14)	0.0229 (7)	
H14	−0.1179	0.1002	0.5213	0.027*	
C15	−0.09263 (16)	0.0552 (4)	0.44471 (14)	0.0244 (8)	
H15	−0.1287	−0.0045	0.4346	0.029*	
C16	−0.04548 (17)	0.0783 (4)	0.40862 (14)	0.0263 (8)	
C17	−0.0465 (2)	−0.0028 (6)	0.35445 (15)	0.0493 (12)	
H17A	−0.0384	0.0838	0.3273	0.074*	
H17B	−0.0869	−0.0539	0.3481	0.074*	
H17C	−0.0149	−0.0913	0.3526	0.074*	

### Atomic displacement parameters (Å^2^)

**Table:**

	*U*^11^	*U*^22^	*U*^33^	*U*^12^	*U*^13^	*U*^23^
Cl1	0.0266 (5)	0.0330 (5)	0.0333 (5)	0.0062 (4)	0.0064 (4)	−0.0064 (4)
Cl2	0.0411 (6)	0.0177 (4)	0.0357 (5)	−0.0001 (4)	0.0043 (4)	−0.0053 (4)
Cl3	0.0293 (5)	0.0327 (5)	0.0184 (4)	−0.0093 (4)	0.0004 (3)	0.0037 (3)
Cl4	0.0211 (4)	0.0374 (5)	0.0245 (4)	−0.0040 (4)	−0.0045 (3)	0.0029 (4)
P1	0.0188 (4)	0.0146 (4)	0.0137 (4)	0.0009 (4)	0.0004 (3)	−0.0004 (3)
P2	0.0245 (5)	0.0173 (4)	0.0192 (4)	0.0029 (4)	0.0014 (3)	−0.0034 (3)
P3	0.0199 (4)	0.0200 (4)	0.0151 (4)	−0.0005 (4)	−0.0005 (3)	0.0000 (3)
O1	0.0211 (12)	0.0192 (12)	0.0161 (11)	−0.0042 (10)	0.0013 (9)	0.0005 (9)
N1	0.0233 (15)	0.0169 (14)	0.0182 (14)	0.0053 (12)	−0.0021 (11)	−0.0008 (11)
N2	0.0315 (17)	0.0241 (15)	0.0174 (14)	0.0053 (13)	−0.0010 (11)	−0.0054 (12)
N3	0.0222 (15)	0.0169 (14)	0.0159 (13)	0.0050 (12)	−0.0021 (11)	0.0026 (11)
N4	0.0170 (14)	0.0163 (14)	0.0152 (12)	−0.0027 (11)	0.0010 (10)	−0.0001 (10)
N5	0.0297 (16)	0.0186 (14)	0.0179 (13)	−0.0036 (13)	−0.0055 (11)	0.0005 (11)
C1	0.0191 (17)	0.0131 (16)	0.0189 (15)	0.0029 (13)	0.0002 (12)	0.0018 (12)
C2	0.0201 (17)	0.0192 (17)	0.0199 (16)	−0.0004 (14)	0.0011 (13)	0.0006 (13)
C3	0.030 (2)	0.0240 (18)	0.0185 (16)	0.0026 (15)	−0.0009 (13)	0.0006 (13)
C4	0.032 (2)	0.031 (2)	0.0212 (17)	0.0033 (16)	−0.0050 (14)	−0.0046 (14)
C5	0.0244 (18)	0.0215 (18)	0.0286 (18)	−0.0027 (16)	−0.0045 (14)	−0.0024 (14)
C6	0.0216 (17)	0.0159 (17)	0.0233 (16)	0.0003 (14)	−0.0043 (13)	0.0003 (13)
C7	0.0166 (17)	0.0177 (18)	0.0295 (18)	−0.0027 (13)	0.0016 (13)	−0.0011 (14)
C8	0.0221 (17)	0.0166 (16)	0.0223 (16)	−0.0005 (15)	0.0031 (13)	0.0002 (13)
C9	0.0198 (17)	0.0139 (16)	0.0164 (15)	0.0033 (13)	−0.0017 (12)	−0.0019 (12)
C10	0.0180 (16)	0.0122 (15)	0.0191 (15)	0.0029 (13)	0.0032 (12)	0.0007 (12)
C11	0.0161 (15)	0.0161 (15)	0.0118 (13)	0.0012 (13)	−0.0002 (11)	0.0025 (12)
C12	0.0187 (17)	0.0129 (16)	0.0235 (16)	0.0020 (14)	−0.0044 (13)	0.0027 (13)
C13	0.0205 (17)	0.0118 (15)	0.0225 (16)	0.0012 (14)	0.0006 (13)	0.0000 (13)
C14	0.0191 (17)	0.0146 (16)	0.0349 (19)	0.0023 (14)	0.0019 (14)	0.0056 (14)
C15	0.0239 (18)	0.0155 (16)	0.0337 (19)	−0.0032 (15)	−0.0093 (15)	0.0058 (14)
C16	0.035 (2)	0.0184 (18)	0.0251 (18)	−0.0069 (16)	−0.0101 (15)	0.0037 (14)
C17	0.069 (3)	0.054 (3)	0.025 (2)	−0.034 (2)	−0.003 (2)	−0.0021 (19)

### Geometric parameters (Å, º)

**Table:**

Cl1—P2	2.0047 (12)	C6—C5	1.419 (4)
Cl2—P2	1.9972 (13)	C6—C7	1.420 (5)
Cl3—P3	2.0165 (12)	C7—C8	1.358 (4)
Cl4—P3	1.9902 (12)	C7—H7	0.9500
P1—O1	1.580 (2)	C8—H8	0.9500
P1—N1	1.596 (3)	C9—C8	1.407 (4)
P1—N3	1.598 (3)	C10—C1	1.436 (4)
P1—N4	1.665 (3)	C10—C9	1.360 (4)
P2—N1	1.571 (3)	C11—C10	1.511 (4)
P2—N2	1.580 (3)	C11—H11A	0.9900
P3—N2	1.581 (3)	C11—H11B	0.9900
P3—N3	1.571 (3)	C12—N5	1.339 (4)
O1—C9	1.399 (4)	C12—C13	1.383 (4)
N4—C11	1.483 (4)	C13—C14	1.389 (5)
N4—C12	1.416 (4)	C13—H13	0.9500
C1—C2	1.417 (4)	C14—C15	1.380 (5)
C1—C6	1.415 (4)	C14—H14	0.9500
C2—C3	1.369 (4)	C15—C16	1.381 (5)
C2—H2	0.9500	C15—H15	0.9500
C3—H3	0.9500	C16—N5	1.348 (4)
C4—C3	1.407 (5)	C16—C17	1.502 (5)
C4—H4	0.9500	C17—H17A	0.9800
C5—C4	1.370 (5)	C17—H17B	0.9800
C5—H5	0.9500	C17—H17C	0.9800
			
O1—P1—N1	108.71 (14)	C1—C6—C7	119.4 (3)
O1—P1—N3	102.53 (13)	C5—C6—C7	120.9 (3)
O1—P1—N4	102.37 (12)	C6—C7—H7	119.4
N1—P1—N3	117.31 (14)	C8—C7—C6	121.3 (3)
N1—P1—N4	110.87 (14)	C8—C7—H7	119.4
N3—P1—N4	113.47 (14)	C7—C8—C9	118.2 (3)
Cl2—P2—Cl1	100.69 (5)	C7—C8—H8	120.9
N1—P2—Cl1	108.08 (11)	C9—C8—H8	120.9
N1—P2—Cl2	110.98 (11)	O1—C9—C8	114.7 (3)
N1—P2—N2	119.09 (15)	C10—C9—O1	121.0 (3)
N2—P2—Cl1	108.95 (12)	C10—C9—C8	124.2 (3)
N2—P2—Cl2	107.47 (12)	C1—C10—C11	119.5 (3)
Cl4—P3—Cl3	100.03 (5)	C9—C10—C1	117.8 (3)
N2—P3—Cl3	108.10 (12)	C9—C10—C11	122.7 (3)
N2—P3—Cl4	108.11 (11)	N4—C11—C10	115.8 (2)
N3—P3—Cl3	109.12 (11)	N4—C11—H11A	108.3
N3—P3—Cl4	109.92 (11)	N4—C11—H11B	108.3
N3—P3—N2	119.75 (14)	C10—C11—H11A	108.3
C9—O1—P1	124.78 (19)	C10—C11—H11B	108.3
P2—N1—P1	121.72 (17)	H11A—C11—H11B	107.4
P2—N2—P3	119.79 (17)	N5—C12—N4	113.8 (3)
P3—N3—P1	120.78 (17)	N5—C12—C13	123.8 (3)
C11—N4—P1	123.9 (2)	C13—C12—N4	122.4 (3)
C12—N4—P1	118.4 (2)	C12—C13—C14	117.3 (3)
C12—N4—C11	116.5 (2)	C12—C13—H13	121.3
C12—N5—C16	117.9 (3)	C14—C13—H13	121.3
C6—C1—C2	118.2 (3)	C13—C14—H14	120.2
C6—C1—C10	119.2 (3)	C15—C14—C13	119.5 (3)
C2—C1—C10	122.6 (3)	C15—C14—H14	120.2
C1—C2—H2	119.5	C14—C15—C16	119.3 (3)
C3—C2—C1	120.9 (3)	C14—C15—H15	120.4
C3—C2—H2	119.5	C16—C15—H15	120.4
C2—C3—C4	120.9 (3)	N5—C16—C15	121.9 (3)
C2—C3—H3	119.6	N5—C16—C17	116.3 (3)
C4—C3—H3	119.6	C15—C16—C17	121.8 (3)
C3—C4—H4	120.2	C16—C17—H17A	109.5
C5—C4—C3	119.6 (3)	C16—C17—H17B	109.5
C5—C4—H4	120.2	C16—C17—H17C	109.5
C4—C5—C6	120.7 (3)	H17A—C17—H17B	109.5
C4—C5—H5	119.6	H17A—C17—H17C	109.5
C6—C5—H5	119.6	H17B—C17—H17C	109.5
C1—C6—C5	119.7 (3)		
			
N1—P1—O1—C9	−81.1 (3)	C10—C1—C2—C3	179.0 (3)
N3—P1—O1—C9	154.0 (2)	C2—C1—C6—C5	0.4 (5)
N4—P1—O1—C9	36.2 (3)	C2—C1—C6—C7	−179.9 (3)
O1—P1—N1—P2	−123.90 (19)	C10—C1—C6—C5	179.9 (3)
N3—P1—N1—P2	−8.3 (3)	C10—C1—C6—C7	−0.4 (5)
N4—P1—N1—P2	124.32 (19)	C1—C2—C3—C4	1.8 (5)
O1—P1—N3—P3	121.15 (19)	C5—C4—C3—C2	−0.9 (5)
N1—P1—N3—P3	2.2 (3)	C6—C5—C4—C3	−0.2 (5)
N4—P1—N3—P3	−129.23 (18)	C1—C6—C5—C4	0.5 (5)
O1—P1—N4—C11	−22.8 (3)	C7—C6—C5—C4	−179.2 (3)
O1—P1—N4—C12	170.1 (2)	C1—C6—C7—C8	−0.3 (5)
N1—P1—N4—C11	93.0 (3)	C5—C6—C7—C8	179.4 (3)
N1—P1—N4—C12	−74.1 (3)	C6—C7—C8—C9	1.0 (5)
N3—P1—N4—C11	−132.5 (2)	O1—C9—C8—C7	174.6 (3)
N3—P1—N4—C12	60.4 (3)	C10—C9—C8—C7	−1.0 (5)
Cl1—P2—N1—P1	128.10 (17)	C9—C10—C1—C2	179.9 (3)
Cl2—P2—N1—P1	−122.37 (17)	C9—C10—C1—C6	0.4 (4)
N2—P2—N1—P1	3.2 (3)	C11—C10—C1—C2	1.3 (5)
Cl1—P2—N2—P3	−116.35 (18)	C11—C10—C1—C6	−178.2 (3)
Cl2—P2—N2—P3	135.37 (17)	C1—C10—C9—O1	−175.1 (3)
N1—P2—N2—P3	8.2 (3)	C1—C10—C9—C8	0.3 (5)
Cl3—P3—N2—P2	111.53 (18)	C11—C10—C9—O1	3.5 (5)
Cl4—P3—N2—P2	−141.03 (17)	C11—C10—C9—C8	178.9 (3)
N3—P3—N2—P2	−14.2 (3)	N4—C11—C10—C1	−173.4 (3)
Cl3—P3—N3—P1	−116.34 (17)	N4—C11—C10—C9	8.1 (4)
Cl4—P3—N3—P1	134.90 (16)	N4—C12—N5—C16	−175.2 (3)
N2—P3—N3—P1	8.9 (3)	C13—C12—N5—C16	4.0 (5)
P1—O1—C9—C8	154.1 (2)	N4—C12—C13—C14	174.3 (3)
P1—O1—C9—C10	−30.1 (4)	N5—C12—C13—C14	−4.7 (5)
P1—N4—C11—C10	4.4 (4)	C12—C13—C14—C15	1.1 (5)
C12—N4—C11—C10	171.6 (3)	C13—C14—C15—C16	2.9 (5)
P1—N4—C12—N5	2.2 (4)	C14—C15—C16—N5	−3.8 (5)
P1—N4—C12—C13	−177.0 (2)	C14—C15—C16—C17	174.1 (4)
C11—N4—C12—N5	−165.9 (3)	C15—C16—N5—C12	0.4 (5)
C11—N4—C12—C13	15.0 (4)	C17—C16—N5—C12	−177.6 (3)
C6—C1—C2—C3	−1.5 (5)		

### Hydrogen-bond geometry (Å, º)

Cg3 is the centroid of the C8-benzene ring.

**Table:**

*D*—H···*A*	*D*—H	H···*A*	*D*···*A*	*D*—H···*A*
C5—H5···*Cg*2^i^	0.95	2.97	3.746 (4)	140
C7—H7···*Cg*3^i^	0.95	2.82	3.507 (3)	130

Symmetry code: (i) *x*, −*y*−1/2, *z*−1/2.
